# Introducing a new Trusted Research Environment - the Safe Haven Artificial Platform (SHAIP).

**DOI:** 10.23889/ijpds.v7i3.2056

**Published:** 2022-08-25

**Authors:** Katie Wilde, Lesley Anderson, Moragh Boyle, Alan Pinder, Alexander Weir

**Affiliations:** 1 University of Aberdeen; 2 Canon Medical Research Europe

## Objectives

Scotland has an established Trusted Research Environment (TREs) through the Scottish Safe Haven Network. These Safe Havens traditionally service structured datasets, however, researchers increasingly require access to large multi-modal linked datasets that include medical imaging. We therefore introduced an equivalent to access anonymised NHS images and reports.

## Approach

A pan-Scotland collaboration of 15 partners from industry, NHS, and academia, collaborated to design and deploy a Safe Haven equivalent for SMEs and researchers interested in accessing anonymised NHS imaging and reports to allow them to develop, test, and validate AI algorithms for greater patient benefit. Two Safe Haven sites, worked with a leading medical software research and development team, to deliver a secure analytical platform for the research and development of AI for medical applications.

## Results

The Safe Haven Artificial Platform (SHAIP) is designed to support development of sophisticated AI components and has been used by several SME’s to undertake exemplar projects in stroke, breast screening, and x-ray interpretation for limb fracture and chest. These researchers were supported by the Safe Haven’s governance processes. SHAIP became the first research environment to be directly linked to the Scottish National PACS archive. Access was provided to project specific deidentified images using ‘Hidden in plain sight’; a privacy and data-structure preserving technique. The researchers were able to upload virtual-machines, or ‘dockers’, to bring their toolsets to their research workspaces. An annotation capability was provided to support ground-truth development for machine learning.

## Conclusion

The project has shown we can pull large scale medical images and reports into a TRE in such a way NHS Boards are reassured by our methods, creating a safe analytical platform for AI development that will benefit patient care by providing faster, cheaper and more accurate solutions.

